# Traditional vs. modified Ringer lactate-based del Nido cardioplegia impacts on clinical outcomes in patients undergoing coronary artery bypass grafting: Results from a prospective, randomized, blinded trial

**DOI:** 10.1051/ject/2024010

**Published:** 2024-12-20

**Authors:** Naser Kachoueian, Salimeh Janghorban, Farhad Gorjipour, Majid Torkashvand, Mohammad Parsa Mahjoob, Hossein Aslani, Mohamadjavad Mehrabanian, Fazel Gorjipour

**Affiliations:** 1 Shahid Beheshti University of Medical Sciences Tehran 1516745811 Iran; 2 Rajaie Cardiovascular, Medical and Research Center Tehran 1995614331 Iran; 3 Iranian Scientific Society of Extra-Corporeal Technology Tehran 1995614331 Iran; 4 Tehran University Medical Sciences Tehran 1416634793 Iran; 5 Physiology Research Center, Iran University of Medical Sciences Tehran 1449614535 Iran

**Keywords:** Cardioplegia solution, Coronary bypass grafting, Myocardial ischemia, del Nido, Adult cardiac surgery

## Abstract

*Introduction*: Myocardial protection with cardioplegia is a crucial approach to mitigate myocardial damage during coronary bypass grafting surgery (CABG) with cardiopulmonary bypass (CPB). The major component of the del Nido cardioplegia solution, Plasma-Lyte A, is difficult to obtain in Iran due to high cost. The objective of the current study was to study if the lactated Ringer’s solution as the base for del Nido solution (LR DN) usage is a viable option as a substitute for Plasma-Lyte A in adult patients presenting for CABG surgery. *Study design and methods*: The present prospective, randomized, blinded study was performed on 18–75-year-old patients ejection fraction (EF) > 35% undergoing CABG with CPB. Patients were randomly allocated to LR DN (modified del Nido cardioplegia) and PL DN (standard del Nido cardioplegia solution) groups. Serum level of cardiac troponin I (cTnI), the type and dosage of inotrope agents, EF, rate of arrhythmia after clamp removal and lactate level were measured and compared between patients of LR DN and PL DN groups. *Results*: 109 patients were recruited. There were no statistically significant differences between groups for cardiopulmonary bypass times, cardiac enzymes, transfusion requirements, and arterial blood gases. There was no mortality for study patients. Postoperative serum levels of cTnI among patients in the LR DN group was significantly higher than patients of the PL DN group after ICU admission and 24 h post-ICU. Also, more patients needed epinephrine administration in the operating room in the LR DN group (29.8% vs. 11.5%; *p*: 0.019 vs. PL DN group). *Conclusion*: We concluded that the standard del Nido cardioplegia solution offers better myocardial protection compared with Ringer’s lactate-based del Nido cardioplegia in adult patients undergoing CABG with CPB. We recommend using standard del Nido cardioplegia with a PL base for patients presenting for CABG surgery.

## Introduction

Cardiac operations with cardiopulmonary bypass (CPB) have variable impacts on patients’ intraoperative and postoperative physiological status, such as hemodynamic instability, hemorrhage, coagulative disorders, inflammatory factors release, oxidative factors production, edema, systemic inflammatory response syndrome, and organ failure [1, 2]. Myocardial protection during ischemia and reperfusion is one of the main problems during cardiac surgery and the cardioplegia solution has a significant impact on preventing inflammatory responses, ischemia, and reperfusion [3–5].

For several decades, del Nido cardioplegia solutions have been used in both pediatric and adult cardiac operations. Cost-effectiveness, single-dose usage without the need for repeated dosing until 90 min, and shorter pump time are reported as del Nido benefits [6–10]. The time between initial and redosing can cause myocardial impairment due to the risk of myocardial warming and ischemia [11]. Moreover, Plasma-Lyte-A as the primary solution for del Nido cardioplegia is cost prohibited in Iran and most other countries [12]. Kantathut et al in their study in 2017, for the first time, reported Ringer’s lactate as the base solution of del Nido cardioplegia and compared that with blood-based St. Thomas’ cardioplegia. The findings of their study showed that del Nido cardioplegia was associated with more favorable myocardial protection with a lower incidence of arrhythmia and shorter duration of both intensive care unit (ICU) and hospital stay [12]. After an extensive literature review, we were unable to find studies comparing the clinical and laboratory impacts of del Nido and modified Ringer lactate-based del Nido among patients. Since the cost of Plasma-Lyte A solution is approximately nine times higher than the lactated Ringer’s solution in our setting, if this offers the same protection, this will significantly reduce the cost of care for patients. The present study compared these two types of del Nido cardioplegia solutions on myocardial damage markers, and other biochemical and clinical outcomes in patients undergoing cardiopulmonary bypass graft surgery (CABG) with CPB.

## Patients and methods

### Study design

A prospective, randomized, blinded clinical trial was designed for patients undergoing CABG with CPB in Imam Hossein Hospital, Tehran-Iran in 2021. The research ethics committee of Shahid Beheshti University of Medical Sciences approved the study protocol (Ethics committee reference number: IR.SBMU.MSP.REC.1401.023) and all of the study patients signed informed consent. Patients were enrolled if they were 18–75 years old, a candidate for elective CABG with CPB, receiving 3–4 vascular grafts, ejection fraction (EF) greater than 35% and no multi-organ dysfunction. Vein grafts were harvested from the patient’s leg and the left internal mammary artery (LIMA) to left anterior descending (LAD) graft was done for all patients in the study groups. Patients with a history of cardiac surgery and infection in recent months and, a history of diabetes and kidney diseases were excluded.

Study patients were randomly, assigned to the modified del Nido cardioplegia with lactated Ringer’s solution (trial; LR DN) and standard del Nido cardioplegia based on Plasma-Lyte A (control; PL DN) groups, according to random block with random numbers table. Study protocol was registered on the Iranian Registry of Clinical Trials (https://irct.ir/trial/65917; IRCT registration number: IRCT20210825052284N1) before starting patient recruitment. EF, duration of mechanical ventilation, inotrope administration, arrhythmia incidence, and ICU stay were the primary endpoints.

The study participants and researchers, all but perfusionists, were blinded to the intervention.

### Conduct of cardiopulmonary bypass

CPB among participating patients in both groups, was managed by roller pomp (target flow 2.4 L/min/m^2^ of the body surface area; range 2.2–2.6). Heparin was boluses at 300 units/kg, and the activated clotting time (ACT) level was maintained at higher than 480 s. ACT was measured using a Hemochron Signature Elite machine (Werfen, MA, USA). Mild hypothermia (32 °C) was used for all patients and mean arterial pressure was between 60 and 80 was used. Minimum blood pressure was defined as less than 60 mmHg and was managed by increasing CPB flow and/or administering phenylephrine. Blood pressure greater than 80 mmHg was managed by decreasing flow to 2.2 and/or infusion of nitroglycerine. Near Infra-red Spectrometry (NIRS) was used in all cases and lactate was also monitored by arterial blood gas every 45 min. SvO2 is not a standard of practice in our setting. The red blood cell transfusion trigger was set at 21–24% hematocrit, depending on criteria such as age, EF%, and Cerebrovascular baseline (e.g., carotid stenosis). Blood gasses were managed using pH-stat titration technique according to the standards of care in our institution. We used the same techniques and parameter values for blood gas management in all patients in both groups.

A single dose of cardioplegia solution (20 mL/kg; max 1000 mL), modified or standard, was administered antegrade as previously described [9]. The components for standard DN and modified DN based in Ringer’s lactated solution is described in Table 1. Cardioplegia solutions were prepared by the perfusionist under sterile conditions. Only the base solution was different between the two formulations. Sodium bicarbonate was added immediately before use by the perfusionist. The cardioplegia solutions were prepared immediately before administration and either Plasma-Lyte A (Samen Pharmaceutical Company, Mashhad, Iran) or lactated Ringer’s solution-based (modified) del Nido solutions were administered in ratio of 1:4 autologous blood:crystalloid. Compositions were according to the protocol in our setting presented in Table 1.

**Table 1:** del Nido (DN) cardioplegia solution’s components and the modified formula.

	Modified DN	Standard DN solution
Carrier	Ringer’s Lactated solution (Sodium 130 mEq/liter; Potassium 4 mEq/liter; Calcium 3 mEq/liter; Chloride 110 mEq/liter; Lactate (CH3CH(OH)COO−) 28 mEq/liter)	Plasma-Lyte A (sodium 140 mmol/L, potassium 5 mmol/L, magnesium 1.5 mmol/L, chloride 98 mmol/L, acetate 27 mmol/L, gluconate 23 mmol/L), 1000 mL
KCl	26 mEq	26 mEq
NaHCO3	13 mEq	13 mEq
Mannitol	3.26 g	3.26 g
Lidocaine	130 mg	130 mg
Final volume	1100 mL	1100 mL
Blood: carrier ratio	1:4	1:4
pH	6.2 (6.0–7.5)	7.4 (6.5–8.0)
Dosage and administration	Initial arrest by a dose of 20 mL∙kg^−1^ and subsequent 10 mL∙kg^−1^ re-dosing after 90 min	Initial arrest by a dose of 20 mL∙kg^−1^ and subsequent 10 mL∙kg^−1^ re-dosing after 90 min

Solutions were administered with the same protocol and the dose of 20 mL/kg of the body weight (maximum 1000 mL per patient weighing over 50 kg). The delivery temperature of the solution was 4 °C and the system pressure was 100–200 mmHg and the administration flow was 200–300 mL/min.

### Data collection and outcomes

Demographic data including age, sex, body surface area (BSA), and body mass index (BMI), blood pressure, heart rate, blood urea nitrogen (BUN), creatinine, amount of blood product usage, urination and hemofiltration volume on CPB were measured during the study period and recorded into the trial checklist. Primary outcomes were EF (before sternotomy and after weaning from CPB in the operation room), duration of mechanical ventilation, post-surgical arrhythmia incidence, and inotrope administration after surgery. Secondary outcomes were serum level of cardiac troponin (cTnI) and lactate dehydrogenase at the preoperative, after cross-clamp removal, ICU admission, and 24 h after ICU admission. All patient’s background information and clinical outcomes along with vital sign monitoring reports were recorded in the trial checklist.

### Statistical analysis

Study data were entered into the Statistical Package for the Social Sciences (SPSS) (IBM Inc., NY, USA) Ver. 22.0 software for data analysis. The normality of study data was analyzed and mean ± standard deviation and frequency percentages were used to describe quantitative and qualitative variables in normal and median ± interquartile range (IQR) for nonparametric variables. Independent sample t-test statistical test was applied for comparing means between two groups. Mann–Whitney *U* test was applied for comparing quantitative data with non-parametric distribution. The Chi-square test was used for comparing frequencies between groups. *p*-values less than 0.05 was considered significant. The power of analysis was set at 80%.

According to the findings of Kantathut et al., the mean (SD) for ICU stay was 2(1.5) days for the modified del Nido group [12]. Considering a one-day difference between the study groups, *α* = 0.05, statistical power = 0.9 and using the Mann–Whitney test for power analysis, the sample size was determined as equally 51 in each group. GPower 3.1 was applied for the calculation.

## Results

**Figure 1:**
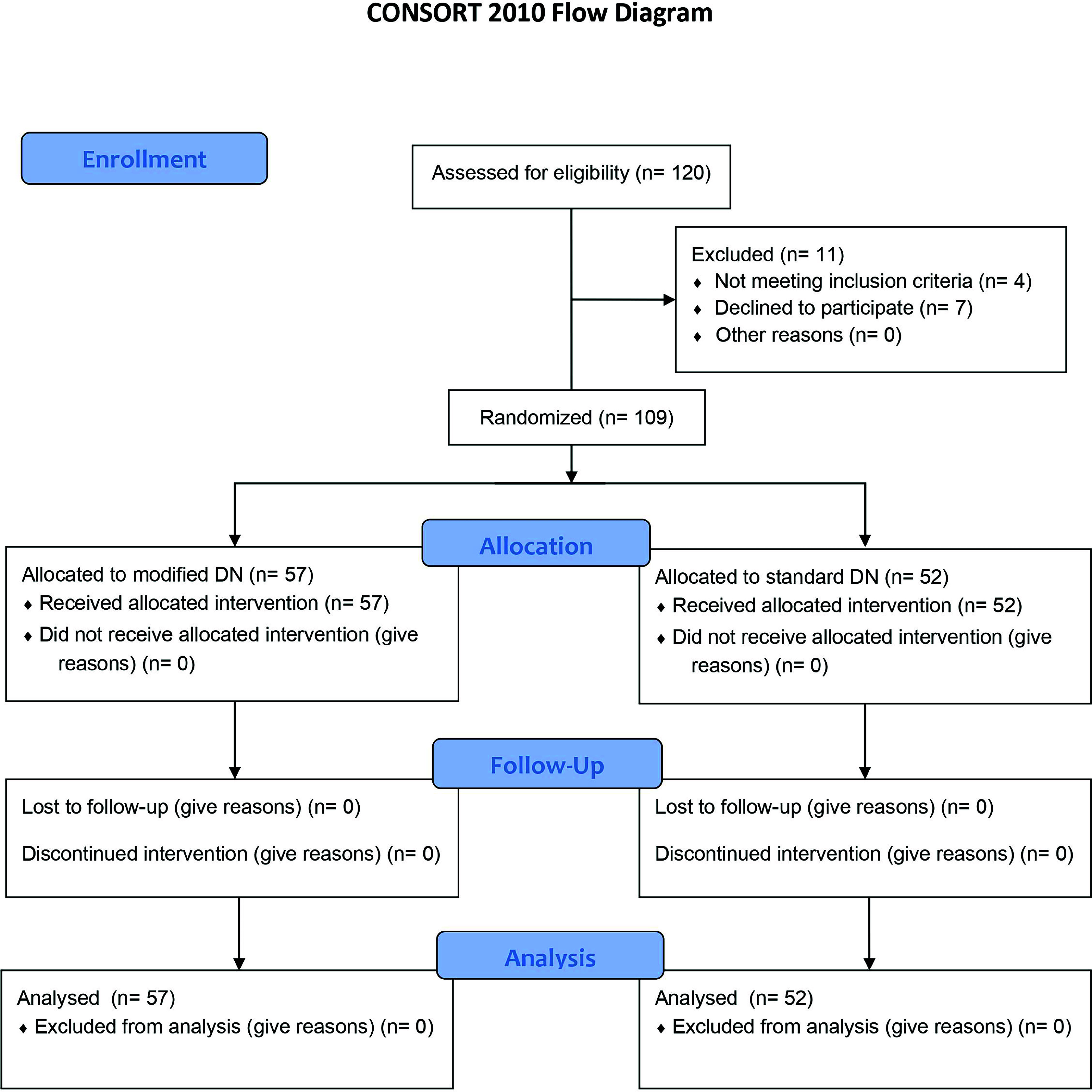
CONSORT flow diagram depicting the process of patient screening, recruitment, and study flow.

The study flow using the Consolidated Standards of Reporting Trials (CONSORT) flow diagram is presented in Figure 1. Finally, from 120 patients screened for the recruitment, 109 patients were recruited. The mean age and body mass index among study participants were 61.41 ± 8.41 years and 27.04 ± 3.84 kg/m^2^ respectively. 80% of the patients were males. Included patients were randomly allocated to LR DN (*n* = 57) and PL DN (*n* = 52) groups. Mean of age and BMI were not significantly different between the two groups. No mortality was observed and arrhythmias occurred in nine patients, four in the LR DN and five in the PL DN (*p*: 0.56). Markers of renal function were similar among patients of both groups in all of the measurements. The incidence of arrhythmia was similar between the two groups (Table 2).

**Table 2:** Frequency distribution of demographic and basal variables among patients of LR DN (modified del Nido) and PL DN groups (conventional del Nido solution).

Groups	LR DN	PL DN	*p*-value
Variables	(Median ± IQR or mean ± SD)	(Median ± IQR or mean ± SD)	
Age (year)	63 ± 11	64 ± 9.5	0.49
BMI (kg/m^2^)	26.22 ± 3.43	27.14 ± 4.45	0.15
BSA (m^2^)	1.80 ± 0.20	1.85 ± 0.23	0.15
Sex (male)	38 (47.5%)	42 (52.5%)	0.13
Cross clamping time (min)	42 ± 16	39.5 ± 12.75	0.06
Mechanical ventilation time (h)	9.42 ± 3.05	9.68 ± 3.31	0.68
Preoperative ejection fraction (%)	50 ± 7.5	50 ± 12.50	0.55
Postoperative ejection fraction (%)	50 ± 8.75	50 ± 10	0.95
ICU stay (h)	48 ± 24	48 ± 24	0.29

BMI: body mass index, BSA: body surface area, CPB: cardiopulmonary bypass, ICU: intensive care unit, IQR: interquartile range. The *p* value was calculated by the student’s *t*-test for data with normal distribution. Frequencies between groups were compared with the Chi-squares test.

The mean of age, BMI, BSA, and male-to-female ratio were similar in patients of the two groups. Meantime to arrest in patients of the LR DN group was significantly lower than patients of the PL DN group (47.50 ± 16.60 vs. 54.59 ± 18.46 s; *p* = 0.04). The mean of the return time to sinus rhythm among patients of the LR DN group was similar to that of patients of the PL DN group (5.30 ± 6.23 vs. 6.01 ± 5.99 min; *p* = 0.55). ICU stay, CPB, aortic cross clamping, frequency of arrhythmia and ejection fraction of patients at pre- and post-operative measurements were similar (Table 2).

**Figure 2:**
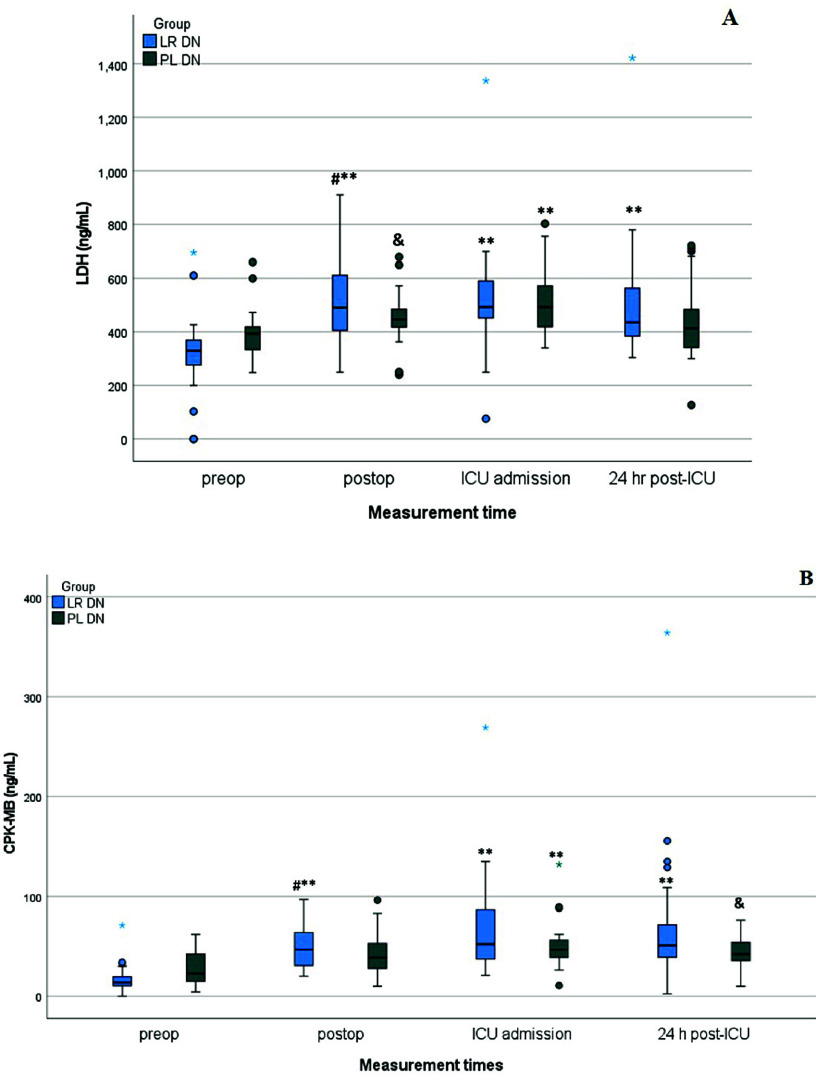
Between-group comparison of the lactate dehydrogenase (LDH) and CPK-MB demonstrated significant differences between two groups at post-operative time. The post-operative value was significantly higher in the Trial group receiving modified del Nido solution based on lactated Ringer’s solution (LR DL) compared with the standard del Nido solution based on Plasma-Lyte A (PL DL). Presented *p*-values were calculated using analysis of covariance (ANCOVA) with Bonferroni adjustment with values for the baseline (preoperative) amounts of LDH (A) and CPK-MB (B) set as covariates to adjust for the differences in the baseline between two groups. # *p* < 0.05 vs. the PL DN group at the same time, & *p* < 0.05 vs. the baseline (preop) in the same group, ** *p* < 0.001 compared with the baseline in the same group.

At the postoperative period, the serum level of cardiac enzymes was not significantly different among patients of both groups. At the ICU entrance time, only the serum level of cTnI among patients of LR DN groups was significantly higher than patients of PL DN group (*p* = 0.005). 24 h after ICU entrance, serum level of creatine phosphokinase-MB (CPK-MB) and cTnI were significantly higher among patients of LR DN group compared with the patients of the PL DN group. Details of comparisons of laboratory tests among patients of LR DN and PL DN groups at different measurement times were presented in the Figure 2 and Table 3.

**Table 3:** Frequency distribution of cardiac enzymes among patients of both groups, LR DN (modified del Nido) and PL DN groups (conventional del Nido solution), at different measurement points.

Groups		LR DN	PL DN	*p*-value
Variables		(mean ± SD)	(mean ± SD)	
Preoperative	CPK (ng/mL)	111 ± 117	101 ± 187.25	0.32
	cTnI (ng/mL)	1.6 ± 4.79	1.52 ± 2.68	0.81
Post-declamp	CPK (ng/mL)	233 ± 314.85	248 ± 112	0.71
	cTnI (ng/mL)	1.85 ± 4.22	1.92 ± 2.82	0.24
ICU admission	CPK (ng/mL)	390 ± 351	383.50 ± 160.75	0.22
	cTnI (ng/mL)	2.20 ± 3.54	2.15 ± 2.60	0.005#
24 h after ICU admission	CPK (ng/mL)	437 ± 215	270.50 ± 199.25	0.18
	cTnI (ng/mL)	2.26 ± 3.90	1.79 ± 1.03	0.03

CPK: creatine phosphokinase, CPK-MB: creatine phosphokinase-MB, LDH: lactate dehydrogenase, cTnI: cardiac troponin I, ICU: intensive care unit, IQR: interquartile range.

# There is a significant difference between groups (*p* < 0.05).

Frequency of blood products transfused were similar between patients of both groups in operation room and ICU (Table 4).

**Table 4:** Frequency distribution of blood product usage among patients of both groups, LR DN (modified del Nido) and PL DN groups (conventional del Nido solution), at different measurement points.

Groups		LR DN		PL DN		*p*-value
Variables		*N*	%	*N*	%	
Operation room	Pack cell	14	24.6	12	23.1	0.856
	Platelet	12	21.1	8	15.4	0.445
	FFP	10	17.5	12	23.1	0.47
	Fibrinogen	0	0	1	1.9	0.293
ICU admission	Pack cell	8	14	11	21.2	0.33
	Platelet	3	5.3	5	9.6	0.384
	FFP	3	5.3	2	3.8	0.724

*n* stands for the frequency of the patients receiving that product in each group and the % for the frequency percent of the patients in each group receiving that product at the corresponding time. ICU: intensive care unit, FFP: fresh frozen plasma.

Values of arterial blood gases was similar between patients of both groups preoperatively, in the operation room and in the ICU (Table 5).

**Table 5:** Values of arterial blood gasses among patients of both groups, LR DN (modified del Nido) and PL DN groups (conventional del Nido solution), at different measurement points.

Groups		LR DN	PL DN	*p*-value
Variables		(mean ± SD)	(mean ± SD)	
Preoperative	PaO_2_ (mm Hg)	343 ± 251	363.50 ± 218.25	0.54
	PaCO_2_ (mm Hg)	39 ± 2	39 ± 2.75	0.84
	HCO_3_ (mm Hg)	27 ± 4	26 ± 34	0.81
Postoperative	PaO_2_ (mm Hg)	281.50 ± 209.50	314 ± 145	0.86
	PaCO_2_ (mm Hg)	39 ± 8	37 ± 5	0.15
	HCO_3_ (mm Hg)	22 ± 3.75	23 ± 2	0.21
ICU admission	PaO_2_ (mm Hg)	157 ± 99	136 ± 70.75	0.24
	PaCO_2_ (mm Hg)	41 ± 9	41 ± 6.50	0.45
	HCO_3_ (mm Hg)	23 ± 3.50	22 ± 4.50	0.96

ICU: intensive care unit, PaO_2_: partial pressure of oxygen; PaCO_2_: partial pressure of carbon dioxide; HCO_3_: bicarbonate, IQR: interquartile range. The fraction of inspired oxygen, FiO2, was 90% according to the standard of practice in our setting.

There was significantly higher epinephrine administration rates in the operation room in the LR DN compared with the PL DN group (29.8% vs. 11.5%; *p*: 0.019). Significant difference in use of other inotropes between two groups was not observed (Table 6).

**Table 6:** Frequency distribution of times of administration of inotropes among patients of both groups, LR DN (modified del Nido) and PL DN groups (conventional del Nido solution), at different measurement points.

Groups		LR DN		PL DN		*p*-value
Variables		*N*	%	*N*	%	
Operation room	Dopamine	1	1.8	0	0	0.337
	Milrinone	1	1.8	0	0	0.337
	Epinephrine	17	29.8	6	11.5	0.019#
	Norepinephrine	0	0.0	0	0.0	–
	Ephedrine	1	1.8	0	0	0.337
	Dobutamine	0	0.0	0	0.0	–
ICU	Dopamine	1	1.8	0	0	0.337
	Milrinone	1	1.8	0	0	0.337
	Epinephrine	9	15.8	6	11.5	0.305
	Norepinephrine	1	1.8	2	3.8	0.323
	Dobutamine	1	1.8	0	0	0.337

*n* stands for the frequency of the patients receiving that product in each group and the % for the frequency percent of the patients in each group receiving that product at the corresponding time. ICU: intensive care unit.

# Significant differences were observed.

There was only one redosing in the modified DN group which was not significantly different from control group (*p* = 0.337). The redosing volume was also half the first dose.

## Discussion

The present study aimed to compare the clinical impact of a modified del Nido (DN) cardioplegia solution based on lactated Ringer’s solution with the standard Plasma-Lyte based DN cardioplegia solution on myocardial function, biochemical parameters, and clinical outcomes in patients undergoing CABG with CPB. Although no significant differences in ejection fraction and duration of ICU stay were observed between the two groups, there were notable differences in biochemical parameters at the postoperative time point. Specifically, the modified DN group exhibited higher levels of CPK-MB, and cTnI compared to the conventional DN cardioplegia group. The elevated cTnI levels in the modified DN group persisted for up to 24 h after ICU admission. Blood gas analysis and transfusion requirements did not differ significantly between the two groups. We also are reporting for the first time that the need for inotrope administration (epinephrine) in the operation room was higher in the patients receiving LR DN rather than PL DN.

In a study by Surabhi et al., a comparison was made between Ringer’s lactate and Plasma-Lyte A as priming solutions in CPB, and it was found that the latter was a superior solution associated with a lower incidence of metabolic acidosis [13]. The researchers reported significantly higher levels of circulatory lactate during and after the operation, as well as 30 min after ICU admission, in the modified DN group compared to the conventional DN group. Similar outcomes were observed in the current study at the postoperative time point, although the differences diminished following ICU admission, likely due to the lower volume of cardioplegia solution used in comparison to the total prime volume reported in the study by Surabhi et al. Furthermore, no differences in systemic acidosis were observed, possibly because the volume of cardioplegia solution used was relatively small compared to the total circulatory volume. Kantathut et al. conducted a study comparing the outcomes of using del Nido cardioplegia with Ringer’s lactate as the base solution vs. blood cardioplegia for cardiac protection [12]. Their findings revealed that the use of the modified DN solution was associated with shorter ICU stays and hospitalizations, as well as a reduced need for vasopressor and inotropic support compared to blood cardioplegia. They also reported a lower incidence of intraoperative atrial fibrillation. Worth noting is that unlike the present study, Kantathut et al. did not compare the modified DN solution based on Ringer’s lactate with the conventional DN solution based on Plasma-Lyte A.

Another study by Talwar et al. compared the standard DN solution with the modified DN solution as cardioplegia in patients under 12 years of age undergoing CPB for surgical correction of tetralogy of Fallot [14]. Their results demonstrated the non-inferiority of the Ringer’s lactate-based del Nido cardioplegia compared to the standard formula. This finding contradicts the results of the current study, which showed the superiority of the standard DN solution compared to the modified DN solution. The differences in findings may be attributed to variations in patient age and corresponding anthropometric characteristics. Adult patients generally have higher body weights, necessitating larger volumes of cardioplegia, which may influence outcomes differently. Additionally, the surgical approaches, cardiac pathologies, and metabolic demand are different in adults compared to children.

It should be noted that Ringer’s lactate solution contains calcium whereas Plasma-Lyte A does not. Intracellular calcium management may therefore be impacted by the higher calcium content of the Ringer’s lactate-based cardioplegia vs. the standard formula.

Kantahut et al. studied the use of LR solution as a base for DN cardioplegia, and reported no differences in clinical and laboratory findings [15]. Their patients were from different cardiac surgery types such as isolated CABG, valve replacement and repair and mixed operations. Our findings are in agreement with Kantahut et al. in terms of clinical outcomes and laboratory findings except for cTnI and epinephrine administration in the operation room. In the LR DN group, cTnI levels were higher after operation and persisted until the 24 h post-operation. This finding is very important and contradicts the findings of Kantahut et al. Since our study population is homogeneous and in isolated CABG patients, the differences could be attributed to the differences in the operation types of patients. Also, we found that epinephrine administration in the operation room after CPB is significantly higher in the LR group which is consistent with the laboratory findings regarding myocardial damage.

While caution is necessary in generalizing the findings of the current study, they do raise concerns about replacing Plasma-Lyte A with Ringer’s lactate in the DN solution during cardiac operations in adults under CPB. Further investigations are needed to fully understand the implications of these findings.

### Study limitations

Interpretation of the findings from the current study is limited by the small sample size. Also, the current study follows up on the patients until 24 h after ICU admission. Studies with longer follow-up time, in the ICU and post-discharge from the hospital, may give better insights into the long-term outcomes of using LR as a substitute with PL as a base solution in the DN cardioplegia.

## Conclusion

In conclusion, based on the significantly higher serum levels of cTnI and the higher need for inotrope at the postoperative and ICU stay measurement points in patients receiving modified DN solution, We recommend using standard del Nido cardioplegia with a base of Plasma-Lyte A for patients presenting for CABG surgery regarding its better myocardial protection in the present study. Further studies are warranted to support or refute the findings of the current research and explore various aspects of using the standard DN solution vs. the modified DN solution.

## Data Availability

Data is available upon request.
